# Better Training for Matrons

**Published:** 1909-02-13

**Authors:** 


### Nursing Administration.

### BETTER TRAINING FOR MATRONS.

### VI.—HOSPITAL ECONOMICS.

Hospital economics is the name given to various subjects connected with the management and up-keep of a hospital. It is a study which English matrons have very little opportunity of entering upon. They learn, of course, a good many facts about the management of hospitals in the course of their training, and in the posts they occupy subsequently; but however keen their powers of observation may be, it is impossible that they can form a clear and comprehensive impression of the intricate matters involved, without systematic instruction. Any well-considered course of training for matrons must embrace a course of hospital economics, and must face the necessity for introducing a far wider range of subjects than the average matron would be called upon to understand in the discharge of her duties. The aim of such a course of study would be to set the student in a position to regard hospital administration as a whole. She will be called on to work in close association with various officials whose duties to a certain extent overlap her own. In some establishments the matron is called on to perform many duties which elsewhere fall to the lot of secretary, steward, or even medical superintendent. Unless she can get a grasp of the whole administration her relations with others in authority will be less easily adjusted, for much which is a matter of course to the instructed appears like causeless interference to a woman blind to everything except what concerns her narrow department.

In addition, then, to the theoretical study of hygiene and physiology, as already suggested, an introduction to the art of teaching, and a period spent in practical work in each department, the student for the matrons' diploma ought to be granted the opportunity of studying the structure of the hospital, its furnishing, the measures needed to keep it in repair and in a sanitary condition, the best means of disposing of refuse and sewage, the arrangements of wards, the admission and discharge of patients, the best division of labour among the members of the staff, the cost of the in-patient and out-patient, the paying ward, and other subjects arising out of these. It would not be difficult in a well-administered hospital to arrange for the delivery of lectures on such matters to selected students among the nurses. Delivered in the hospital they would have the overwhelming advantage of affording practical illustrations of the matter in hand at every point. The medical superintendent could take his class round the hospital, could point to defects in one ward, to improvements in another, and lay the foundation for a sound knowledge of the principles governing hospital structure, without confusing his hearers by any attempt to plunge them into the mysteries of architecture. They could study the ground plan of the drainage system, learn the best methods of purifying old wards and floors, and become thoroughly conversant with ventilation theories and practice. All this could be done without expensive plant, and without deranging the work of anyone in the institution. It is deplorable that it is not done, and that nurses pass away from hospitals where every detail is a triumph of mind over circumstances without learning to appreciate the value of what lies around them, and without the knowledge which would enable them to imitate the things they see.

Finally, not the least important part of the matrons' course would consist of lectures given by the matron on the peculiar duties and responsibilities of that position. On the matron herself must devolve the task of teaching the art of supervision, the management of the training school, and the secret of success in superintendence. If it may be taken for granted that the delivery of lectures on her own responsibilities would prove another tax on an already overtaxed official, it may also be surmised that few matrons would grudge this extra labour for the purpose of fitting pupils who had earned her confidence by years of conscientious work worthily to fill positions of authority in their turn.

The course, as briefly outlined in the foregoing articles, would consist of the following sections:

1. Private study, supervised by a coach, of hygiene, physiology, elementary bacteriology, and nursing subjects, including the history of nursing.
2. Practical instruction in teaching junior probationers, by means of supervision and criticism.
3. Practical work in every department of the hospital under a competent head, including a term of service in the housekeeper's department, in the nurses' home, the linen department, the laundry, in inspection of the domestic staff, etc., etc.
4. A course of lectures on hospital economics, illustrated by inspection of the various departments comprised in the course.
5. Lectures from the matron on the management of the training school and the general duties of superintendence.

This course of training for matrons might be open to probationers who had passed an examination with credit at the end of two years' service and wished to qualify for higher appointments, or it might be reserved for those who had completed their course and taken their certificate. It would not necessarily interfere with the ordinary course of work, for the spells of duty in the household departments could still be alternated, as at present, with nursing duties. In hospitals where opportunities for such a course were presented, there would undoubtedly be many applications from nurses fully trained in other institutions to enter for the two years' additional instruction; and since two years is admittedly a very short time in which to master the subjects suggested, it is not improbable that the effect of introducing the matrons' diploma in suitable institutions would be to prolong the complete training to five or even six years.

